# Acyclovir for ventilator-associated pneumonia refractory to antibiotics and with high viral herpes simplex load: we are not sure

**DOI:** 10.1186/s13054-020-02868-9

**Published:** 2020-05-26

**Authors:** Patrick M. Honore, Aude Mugisha, Luc Kugener, Sebastien Redant, Rachid Attou, Andrea Gallerani, David De Bels

**Affiliations:** ICU Department, Centre Hospitalier Universitaire Brugmann-Brugmann University Hospital, Place Van Gehuchtenplein, 4, 1020 Brussels, Belgium

We read with great interest the recent paper by Schuierer et al., who conclude that acyclovir treatment was associated with a significantly longer time to death in the intensive care unit (ICU), reduced hazard ratio for ICU death, and improved circulatory and pulmonary oxygenation function in patients with ventilator-associated pneumonia (VAP) not responding to antibiotic treatment and with high herpes simplex virus (HSV) load. They suggest testing all patients with a diagnosis of antibiotic refractory VAP for HSV replication in respiratory secretions and considering acyclovir treatment if more than 10^5^ copies/mL are detected [1]. We would like to make some comments. First, this area remains controversial as several prospective studies have failed to show an increase in mortality associated with HSV infection [2] and the only prospective therapeutic study is limited by small sample size and prophylactic (rather than treatment) dosing [3]. Also, as clinicians, we need to take into account the side effects of the drugs we prescribe and indeed acyclovir is not a benign drug. Nephrotoxicity is the most important side effect of acyclovir, with an overall incidence of AKI of 13%, half of which are KDIGO grade 2/3 [4]. AKI has been found to occur more frequently in patients with pre-existing chronic kidney disease (CKD), diabetes, and in patients treated with higher daily doses of acyclovir [4]. Despite its importance, the acyclovir dose that patients received was not reported in this study [1]. Furthermore, for a study of a drug with known renal toxicity, there is a striking paucity of information regarding renal parameters. There is an upward trend in the incidence of dialysis in those receiving acyclovir, though the difference was not statistically significant, perhaps due to the small number of patients [1]. It should also be noted that acyclovir may be more toxic if given in conjunction with some antibiotics, such as was the case in this study [1]. In a recent study looking at acyclovir-associated AKI, multivariate analysis indicated that the presence of diabetes, concomitant non-steroidal anti-inflammatory drugs (NSAIDs), and vancomycin use were independent risk factors for acyclovir-associated AKI, and higher mortality was observed in AKI patients [5]. Nephrotoxicity associated with IV acyclovir is common and necessitates renal function monitoring. Randomised control trials with more comprehensive data on dose and renal parameters are needed before recommendations regarding acyclovir treatment in the setting of VAP can be made.

## Authors’ response

Reinhard Hoffmann^2,3^, Lukas Schuierer^1,2,3^

1. TUM Graduate School, Technical University of Munich (TUM), Germany

2. Institute for Laboratory Medicine and Microbiology, University Hospital Augsburg, Germany

3. Faculty of Medicine, Augsburg University, Germany

We appreciate the interest of professor Honore and colleagues in our recent publication and thank for their qualified comments.

First, we totally agree that the subject remains controversial and that larger, prospectively randomised trials are needed. To complete our picture, we re-analysed the data and compared 30-day-mortality in our patient cohort. We could confirm our findings by demonstrating that acyclovir significantly reduced 30-day-mortality in high load patients, only (Table 1). Thus, in the absence of higher quality data, we think that our study may add a small but significant piece to the larger puzzle and may aid clinicians in specific situations.

**Table 1:** Evaluation of 30-day mortality

	All patients			Low viral load (10^3^-10^5^ copies/mL)			High viral load (>10^5^ copies/mL)		
	Untreated *n* = 24	Treated *n* = 65	*p*	Untreated *n* = 14	Treated *n* = 16	*p*	Untreated *n* = 10	Treated *n* = 49	*p*
30-day mortality	14 (58%)	27 (42%)	0.231	6 (43%)	6 (38%)	1	8 (80%)	21 (43%)	**0.042**

*p*-values were calculated using the Fisher’s exact test (significant values are indicated in bold: <0.05)

Next, we also agree that possible side effects have to be considered before prescribing any drug to any ICU patient. Therefore, we strictly focused on patients with a high likelihood of viral disease. In our cohort, a maximum dose of 9 mg/kg [median, IQR 7–11] was administered three times daily. Total acyclovir treatment duration of surviving patients was 10 days [median, IQR 6.5–14]. The decision to treat patients with parenteral acyclovir and possible dose reductions in response to worsening renal function was left to the treating clinicians. Table 2 shows the daily acyclovir doses and important renal parameters over the course of antiviral treatment. If at all, only a slight decrease in daily urine volume and a slight increase in the number of patients with estimated GFR < 60 ml/min/1.73 m^2^ and a slight increase in creatinine values are evident. We suggest that antiviral treatment may have an impact on renal function, but at least in our cohort, this is not clinically significant.

**Table 2:** Renal function of antivirally treated patients (*n* = 59)

Antiviral treatment	Daily dose of aciclovir (mg)	Daily urine volume (ml)	Estimated GFR <60 ml/min/1.73m2	Serum creatine (mg/dl)
Day 0	1000 [750 -1500]; *n* = 58	2300 [1550-3175]; *n* = 59	41.1 [21.1-50.1]; *n* = 19	1.01 [0.66-1.23]; *n* = 55
Day 1	2250 [1500-2250]; *n* = 58	2700 [1800-3600]; *n* = 59	40.5 [22.4-47.3]; *n* = 22	0.93 [0.67-1.39]; *n* = 57
Day 2	2100 [1400-2250]; *n* = 57	2404 [1435-3438]; *n* = 58	44.7 [27.8-51.7]; *n* = 25	1.04 [0.68-1.35]; *n* = 57
Day 3	1850 [1150-2250]; *n* = 52	2620 [1390-3813]; *n* = 52	41.6 [31.7-47.4]; *n* = 22	0.96 [0.65-1.50]; *n* = 51
Day 4	2000 [1000-2250]; *n* = 45	2900 [2000-3350]; *n* = 45	40.3 [20.3-45.2]; *n* = 17	0.97 [0.69-1.42]; *n* = 44
Day 5	2000 [1260-2250]; *n* = 36	3220 [2075-4293]; *n* = 36	41.3 [38.4-50.1]; *n* = 17	1.04 [0.67-1.46]; *n* = 37
Day 6	2250 [1500-2250]; *n* = 34	2725 [1928-3868]; *n* = 34	42.0 [39.5-48.0]; *n* = 14	0.97 [0.66-1.44]; *n* = 33
Day 7	2125 [1500-2250]; *n* = 30	2800 [2075-4075]; *n* = 30	42.3 [37.8-48.8]; *n* = 12	0.84 [0.62-1.35]; *n* = 31

Data from clinical charts and laboratory information system is restricted to its availibility (not for patients who died) and to the phase of antiviral treatment. One gangciclovir treated patient was excluded for calculation of the daily acyclovir dose. The different variables are shown as medians, followed by the interquartile ranges in brackets [IQR] and finally added by the number (n) of values availibe. Estimated glomerular filtration rate (GFR) was calculated by MDRD Equation and only listed if the estimated GFR was below 60 ml/min/1.73m2

This is well in line with published data. In a recently published trial, IV acyclovir treatment at 5 mg/kg TID was considered safe, with no difference in renal failure, creatinine increase, and renal replacement therapy rates compared to the placebo group [6]. Richelsen et al. described nephrotoxicity in 5.1–10.5% of patients receiving 10 mg/kg acyclovir TID, which was fully reversible [7]. Moreover, older studies already showed that nephrotoxicity was usually reversible and could be minimised by slow infusion and adequate hydration [8]. Thus, we fully agree that the administration of high acyclovir doses may impose some risk of renal failure, which has to be weighed against the probable survival benefit in patients with otherwise unexplained VAP and high HSV load in the lower respiratory tract.

Sincerely,

Reinhard Hoffmann

Lukas Schuierer

## Data Availability

Not applicable.

## Abbreviations

ICU: Intensive care unit
VAP: Ventilator-associated pneumonia
HSV: Herpes simplex virus
AKI: Acute kidney injury
KDIGO: Kidney disease improvement global outcomes
CKD: Chronic kidney disease
NSAIDs: Non-steroidal anti-inflammatory drugs
